# TD‐NMR Structural Profiling of Meat and Plant‐Based Meat Analog Burgers

**DOI:** 10.1111/1750-3841.70376

**Published:** 2025-07-03

**Authors:** Zeev Wiesman, Moshe Hai Azachi, Tatiana Oshether

**Affiliations:** ^1^ Phyto‐Lipid Biotech Lab (PLBL), Department of Biotechnology Engineering, Faculty of Engineering Sciences Ben Gurion University of the Negev Beer Sheva Israel

**Keywords:** food quality control, meat burger, plant‐based meat analogs, structural analysis, TD‐NMR relaxation

## Abstract

The functional properties of food are closely linked to its atomic, molecular, and microstructural characteristics. This study evaluates the potential of time‐domain nuclear magnetic resonance (TD‐NMR) relaxometry as a non‐destructive technique for profiling the internal microstructure of Soy‐based plant‐based meat analogs (PBMA) compared to Angus beef burgers. TD‐NMR results were supported by measurements of water content, water release, and morphological analysis using visual and confocal microscopy. Microscopy revealed clear structural differences: Soy‐PBMA burgers exhibited a gel‐like, porous matrix, while Angus beef burgers showed compact, fibrous bundles characteristic of natural muscle tissue. Moisture analysis indicated lower total water content in Soy burgers, while water release tests demonstrated higher water‐holding capacity in Angus burgers, suggesting stronger protein–water interactions. TD‐NMR relaxation data reflected these structural distinctions. T₁ recovery times were longer in Angus burgers, while T₂ relaxation times were shorter and more narrowly distributed, consistent with tightly packed myofibrillar proteins. In contrast, Soy‐PBMA burgers exhibited broader T₂ distributions, reflecting a looser, more porous plant‐protein matrix. One‐dimensional (1D) and two‐dimensional (2D) T₁–T₂ spectral fingerprinting further highlighted differences in proton relaxation behavior, particularly between the myofibrillar proteins of meat and the globular proteins of plant‐based formulations. These findings confirm TD‐NMR relaxometry as a rapid, reliable, and non‐destructive method for differentiating the internal structure of meat and plant‐based products. This approach offers valuable insights for product development, quality assurance, and the design of next‐generation plant‐based meat alternatives.

## 1 Introduction

Plant‐based meat analog (PBMA) products have gained increasing consumer interest in recent years. The key to their success lies in their ability to closely mimic the sensory properties of real meat (Dekkers et al. 2016). However, fundamental differences in the chemical composition and morphological structure between PBMAs and meat present challenges in replicating the desired meat texture (Abdullah et al. 2024).

Meat primarily consists of skeletal muscle along with associated fat and connective tissue. It is composed of approximately 60%–75% water and about 20% protein, mainly myofibrillar proteins such as actin and myosin, and connective stromal proteins including collagen and elastin (Edwards et al. 2023; Konrad et al. 2024). These proteins are arranged in bundles of muscle fibers, which are surrounded by connective tissue, contributing to toughness. The alignment and length of these fibers significantly influence the texture and mouthfeel of meat. Meat also contains about 3% fat, along with small amounts of carbohydrates and minerals (Cobos and Díaz 2015). It is well recognized that muscle structure and composition play a crucial role in determining meat quality (Listrat et al., 2020).

Commercially available PBMA products generally contain 50%–65% water and 10%–20% vegetable globular proteins, such as glycinin and β‐conglycinin (Kyriakopoulou et al. 2021). These proteins naturally have a compact, spherical shape, which contrasts with the long, fibrous proteins found in muscle tissue. PBMAs also contain approximately 3% plant‐based fats, small amounts of cellulosic carbohydrates as binding agents, and various flavor‐enhancing additives. Soy protein isolate is a common raw material in PBMA production due to its favorable properties and cost‐effectiveness. Often, a mixture of defatted soy flour and wheat starch containing gluten is also used (Ahmad et al. 2022; Dubey et al. 2024; Konrad et al. 2024).

These globular proteins maintain their structure through hydrogen bonds, disulfide bridges, and hydrophobic interactions. In their native state, they are water‐soluble and not naturally fibrous or aligned like muscle proteins (Kyriakopoulou et al. 2021). To mimic meat's fibrous texture, plant proteins undergo extrusion processing, where heat, moisture, and pressure denature the globular proteins. This process unfolds the proteins, allowing them to hydrate, realign, and cross‐link into a fibrous‐like structure intended to resemble meat in both appearance and texture. However, in most PBMA products, the fibrous texture remains less satisfactory (Dekkers et al. 2018; Konrad et al. 2024).

The perception of food texture is directly related to how its structure deforms during handling and consumption (Chen and Rosenthal 2015). Texture changes are linked to structural modifications caused by ingredient interactions during food preparation (De Marchi et al. 2021).

The relationship between water and meat structure is fundamental to understanding meat texture, juiciness, and overall quality (Bertram et al. 2002; Ahmad et al. 2022; Hu et al. 2024). Water in meat is not merely a filler; it interacts closely with muscle proteins, connective tissue, and fat, influencing the physical structure and functional properties of the product (Dekkers et al. 2016). Water in meat exists in three primary forms, classified by how tightly it associates with tissue components (Schreuders et al. 2021): Bound water, tightly bound to charged groups on protein surfaces (e.g., actin, myosin). Intracellular immobilized water, located within muscle structures such as myofibrils. Free water, found in extracellular spaces or between muscle fibers. The state of water reflects the integrity and functional quality of meat tissue. Similarly, the relationship between water and the structure of soybean‐based plant burgers is critical to texture, mouthfeel, and product stability (Dekkers et al. 2016). Water plays both physical and chemical roles in shaping the plant‐based matrix, interacting with soy proteins, fibers, starches, and other components. In plant‐based burgers, water also exists in different physical states: Bound water, strongly bound to soy proteins and polysaccharides, maintains matrix hydration. Immobilized water, trapped within protein or fiber networks, contributes to texture. Free water, present in the voids or pores between protein vesicles encapsulating oils (Dekkers et al. 2018).

Currently, technologies used to characterize meat and PBMAs include mechanical testing, spectroscopic methods, and imaging techniques (Schreuders et al. 2021). However, as noted by Dekkers et al. (2018), dedicated analytical methods specifically tailored to PBMAs are still limited.

Time domain nuclear magnetic resonance (TD‐NMR) proton relaxation is widely used for food quality analysis (Hills 2006; Van Duynhoven et al. 2010; Guthausen 2016; Colnago et al. 2021). TD‐NMR is a powerful, non‐destructive technique for structural profiling of both meat and plant‐based meat analog (PBMA) burgers, particularly in assessing water and fat mobility, distribution, and their interactions within the food matrix (Bertram et al. 2002). It provides valuable insights into internal microstructure, moisture states, and textural attributes. More specifically, TD‐NMR sensors are essential for structural analysis of meat and Soy‐PBMA products because they enable rapid, non‐destructive, and highly informative measurements of internal composition and structure—especially regarding water and fat distribution, mobility, and binding state—which directly influence critical quality attributes such as texture, juiciness, stability, and shelf life (Khan et al. 2022).

TD‐NMR measures the relaxation behavior of hydrogen nuclei (primarily from water and fat) following excitation by a magnetic pulse (Guthausen 2016). Two key relaxation times are observed: T₁ (spin‐lattice relaxation time)—related to the content and physical state of water and fat. T₂ (spin‐spin relaxation time)—reflects water mobility, which correlates with texture, water‐holding capacity, and protein structure. Additionally, TD‐NMR can detect phase transitions and diffusion processes that provide essential information about the physical properties of the sample (Krishnan 2019; Acri et al. 2021).

The main advantages of TD‐NMR sensors include: First, non‐destructive and rapid analysis: Unlike traditional histological or chemical methods, TD‐NMR requires no sample destruction or extensive preparation, making it ideal for in‐line quality control and process monitoring during manufacturing. Second, sensitivity to water and fat states: TD‐NMR detects hydrogen nuclei (¹H), enabling detailed analysis of water phases (bound, immobilized, and free) as well as fat content and physical state. Third, differentiation of matrix interactions: TD‐NMR can distinguish between interactions such as water binding to myofibrillar proteins in meat and water interactions within gel‐like or fibrous networks in plant‐based meat analogs. This reveals how effectively the plant matrix mimics real meat at the molecular level. Fourth, correlation with texture and juiciness: Water mobility measured by TD‐NMR closely correlates with sensory attributes—less free water typically corresponds to a firmer texture, while more mobile water relates to juiciness perceived during chewing. Fifth, monitoring processing effects: TD‐NMR can track structural changes caused by cooking, freezing/thawing, aging/storage, and ingredient reformulation (e.g., fat or binder replacement). Finally, applicability to both meat and plant‐based products: The technique is versatile and suitable for a wide range of product types.

In this study, we aimed to explore the feasibility of using TD‐NMR relaxometry methodologies for microstructure profiling of Soy‐PBMA burgers compared to Angus beef burgers. One‐dimensional (1D) and two‐dimensional (2D) T_1_–T_2_ spectral fingerprinting, as well as self‐diffusion coefficient (*D*) measurements, were performed on each burger type to facilitate comparison. Water content, distribution, and visual and microscopic morphological analyses were conducted to support and validate the TD‐NMR findings.

## 2 Materials and Methods

### 2.1 Materials

The analyses were conducted on samples of animal‐based Black Angus beef burgers (sourced from Loup River Beef, Nebraska, USA) and Tivall Vegan Soybean PBMA burgers (produced by Osem, Israel), both purchased from local suppliers. The Angus burger primarily consists of beef meat with water, proteins, and fats, and some products may include fillers such as additional water, salt, wheat fiber, black pepper, and the preservative E‐223. The standard production process for Angus burgers involves trimming and grinding the meat, followed by mixing and blending all ingredients. The Tivall Vegan Soybean Burger contains textured vegetable proteins (water, soy protein concentrate, and wheat starch containing gluten), water, vegetable oils (rapeseed and sunflower), corn starch, the thickener methyl cellulose, salt, flavorings, maltodextrin, barley malt extract, yeast extract, spices (black pepper, cumin, rosemary), baking powder (diphosphates, sodium carbonates), and rice flour. All chemicals and reagents used in this study were of analytical grade.

### 2.2 Low‐Field NMR Analysis

The ¹H LF‐NMR measurements were carried out on a Maran bench‐top pulsed NMR analyzer (Resonance Instruments, Witney, UK) with a permanent magnet and an 18 mm probe head operating at 23.4 MHz. Initially, the sample was stabilized at 40°C and then equilibrated inside the instrument.

The 1D spectrum of T₂ (spin‐spin) relaxation time constants were generated using a Carr–Purcell–Meiboom–Gill (CPMG) pulse sequence (Carr and Purcell 1954; Meiboom and Gill 1958). The 1D spectrum of T₁ (spin‐lattice) relaxation time constants was generated using an inversion recovery pulse sequence. The 2D T₁–T₂ cross‐correlation experiments were performed using an inversion recovery‐CPMG sequence, following (Resende et al. 2019, Resende et al. 2020). Signal processing was based on a primal‐dual convex objectives (PDCO) inverse Laplace transform optimization algorithm with *α* = 0.5 (Berman et al. 2013; Campisi‐Pinto et al. 2018, Campisi‐Pinto et al. 2020).

The self‐diffusion measurements were carried out using a 20 MHz minispec bench‐top pulsed NMR analyzer (Bruker Analytic GmbH, Berlin, Germany), equipped with a permanent magnet and a 10 mm temperature‐controlled probe head, following the method described by (Stejskal and Tanner 1965) and (Aghelnejad et al. 2022). Before each measurement, the samples were stabilized at 40°C and then equilibrated inside the instrument. The self‐diffusion coefficient (*D*) was determined using the pulsed‐field gradient spin‐echo (PFGSE) method (Osheter et al. 2022). The pulse sequence was performed with 16 scans, *τ* = 7.5 ms, and a recycle delay of 6 s. Typical gradient parameters were *Δ* = 7.5 ms, *δ* = 0.5 ms, time between the 90° pulse and the first gradient pulse = 1 ms, and *G* = 1.6 T/m. Each reported value of the self‐diffusion coefficient (*D*) represents the average of ten measurements.

### 2.3 Confocal Laser Scanning Microscope Images

Imaging was performed using a Zeiss LSM880 Confocal Laser Scanning Microscope equipped with Super‐Resolution capabilities (Zeiss LSM880 Airyscan). A 63× Zeiss Plan‐Apochromat oil immersion objective lens with a numerical aperture of 1.4 and differential interference contrast (DIC) capabilities was used for image acquisition. The excitation source was an argon laser operating at a wavelength of 488 nm (McClements et al. 2021).

Defrosted samples of both Angus beef and Soy‐PBMA burgers were thinly sliced using a sterile scalpel to ensure minimal structural disruption. The slices were then diluted with distilled water at three different ratios (1:10, 1:50, and 1:100, w/v) to assess the optimal concentration for confocal imaging and minimize signal saturation or scattering effects. A small aliquot of each diluted sample was transferred onto a clean glass microscope slide, gently covered with a coverslip, and placed directly onto the Confocal laser scanning microscopy (CLSM) stage for imaging. CLSM was employed to capture high‐resolution images, with Z‐stack imaging performed to obtain optical sections at multiple depths within the samples.

### 2.4 Water Content Analysis

Samples of raw Angus burgers and raw Soy‐PBMA burgers were first weighed and then dried in an oven at 100°C for 24 h. After drying, the samples were weighed again, and the percentage of water loss was used to calculate the total water content using the gravimetric method ([wet/dry] × 100), as reported by Kolar (1992).

Additional samples of Angus meat and Soy‐PBMA burgers were stabilized at 40°C, weighed, and placed in centrifuge tubes fitted with a plastic filter. These samples were centrifuged at a low speed of 1500 rpm for 10 min to minimize structural deformation (Barbut, 2024). After centrifugation, the samples were weighed again, and the percentage of released water was determined as an indicator of bound water capacity ([wet/dry] × 100).

The self‐diffusion coefficient (*D*) of each burger type was measured before the water‐release treatments (oven drying and centrifugation). Fifteen samples of each burger type were tested, with ten measurements taken per sample. All data are presented as mean ± standard deviation (SD).

### 2.5 Database

The database comprises two burger types: Type A—Angus beef burgers, and Type B—Soy‐PBMA burgers. A total of 30 burgers were analyzed, with 15 from each type. Each burger was divided into five pieces, resulting in 150 individual samples (15 × 5 + 15 × 5 = 150; see Supporting Information). TD‐NMR T₁ and T₂ analyses were performed on all samples. Each piece underwent 30 repeated NMR measurements, yielding a total of 4500 datasets (150 × 30 = 4500). Each measurement generated 16,384 time‐resolved data points, represented as decay curves (see Supporting Information), which were processed to produce T₁ and T₂ fingerprint spectra.

### 2.6 Statistical Analysis

**FIGURE 1:**
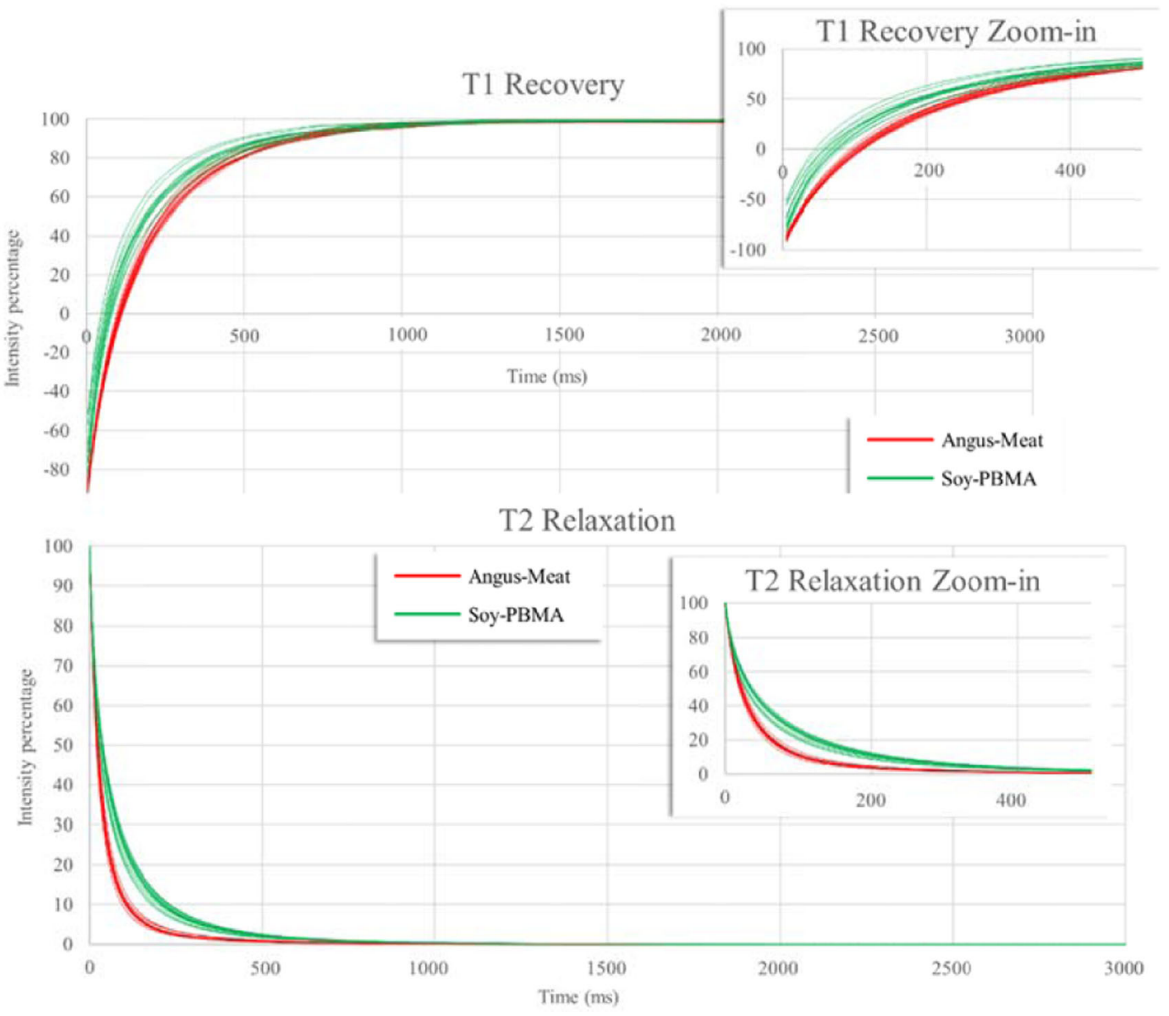
Comparison of fifteen replicates of TD‐NMR measurements showing 1D T₁ recovery curves (top) and T₂ decay curves (bottom) for Angus meat and Soy‐PBMA burger samples. A zoomed‐in view of the most relevant time interval (up to 500 ms) is included in each graph to highlight the early‐phase relaxation behavior.

The database was utilized for all experiments presented in this study. The 1D T₁ recovery and T₂ decay graphs of Angus beef and Soy‐PBMA burgers shown in Figure 1 are based on data from all 15 replicates for each burger type. To capture variability and increase confidence in the results, thirty replicates were used in Figure 4 to generate 2D T₁–T₂ relaxation fingerprinting maps, highlighting the main components (e.g., proteins, oils, and polymers) in Angus beef and Soy‐PBMA burgers.

The total water content and centrifuge water release results presented in Table 1 are based on six replicates per parameter and are expressed as average percentage values ± standard deviation (SD) for both Angus and Soy‐PBMA burgers. The NMR measurements of the self‐diffusion coefficient (*D*) for raw, untreated Angus and Soy‐PBMA burgers before oven heating are based on sixty‐four replicates. All results in Table 1 are presented as mean ± SD.

**TABLE 1:** Comparison of self‐diffusion coefficient *D*, total water content, and bound water content of Angus meat and Soy‐PBMA burgers. Total water content was gravimetrically measured and determined after oven heating ([wet/dry] × 100), and bound water content was obtained by low‐speed centrifugation of raw burger samples. Diffusion coefficients were determined from the same raw samples before oven heating using the PFGSE TD‐NMR method. Values are presented as averages ± standard deviations (SD).

Sample	Diffusion coefficient, *D* (m^2^/s ×10⁻⁹)	Total water content (%)	Bound water content (%)
Angus meat	1.46 ± 0.12	68.9 ± 0.86	13.8 ± 3.3
Soy‐PBMA	1.37 ± 0.05	60.8 ± 0.33	9.3 ± 0.9

## 3 Results

**SCHEME 1:**
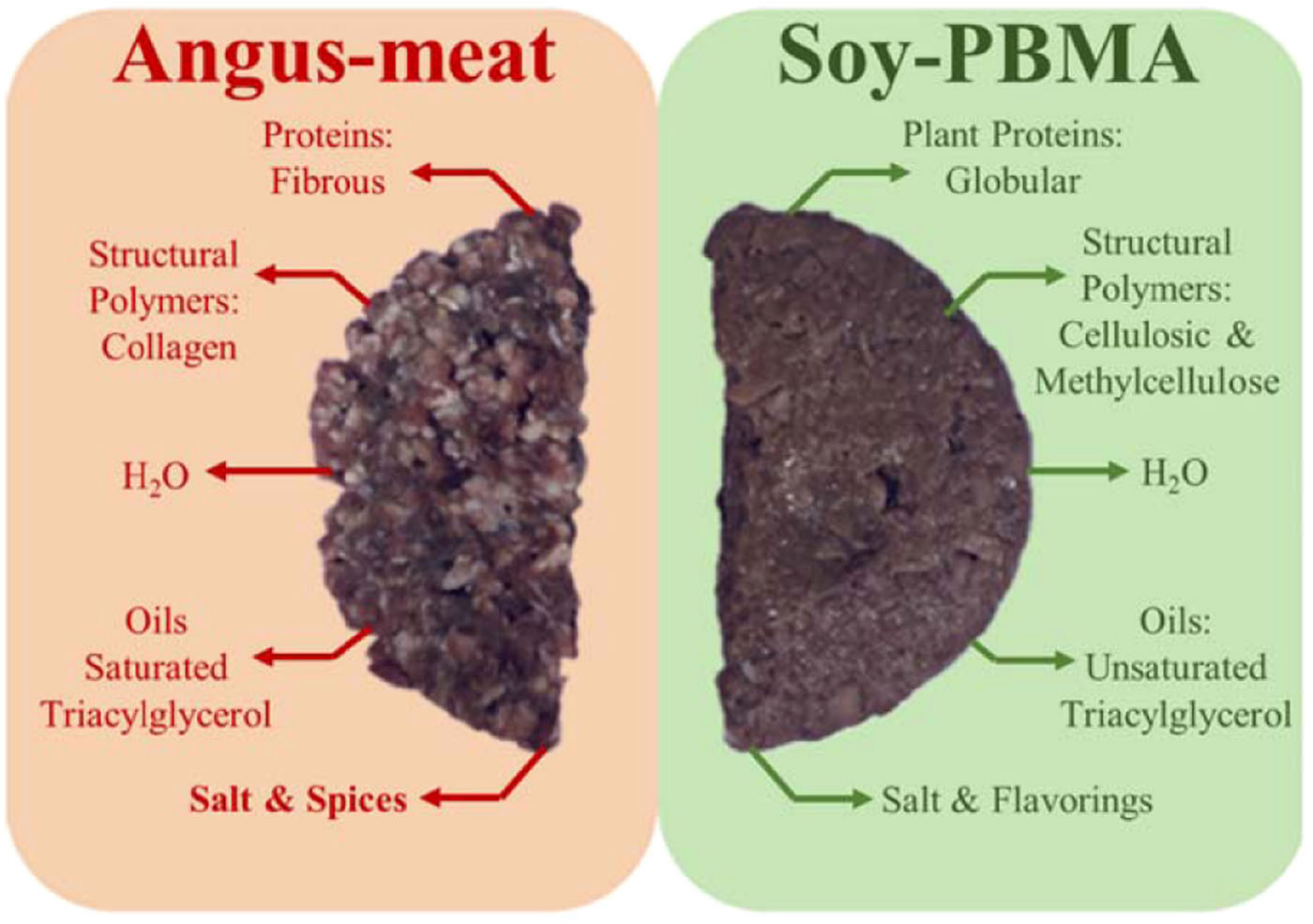
Visual appearance and main structural components of Angus meat and Soy‐PBMA burgers. The Angus meat burger consists of fibrous proteins, structural polymeric collagen polypeptides, water (H₂O), saturated triacylglycerols (TAGs), salt, and spices. The Soy‐PBMA burger contains globular plant proteins, structural polymeric plant cellulose and methyl cellulose fibers, water (H₂O), unsaturated TAGs, salt, and flavorings. Images were obtained specifically for this study at PLBL.

In Scheme 1, we present the typical visual appearance and main chemical components of Angus meat and Soy‐PBMA burgers used in this study. The key differences in structural components—such as proteins, oils, polymers, and binders—between these two types of burgers are highlighted. The Angus meat burger is characterized by a chopped, aggregated structure composed of brown bundles of fibrous proteins and white spots of oils, collagen‐based connective structural polymers, salts, and spices. In contrast, the Soy‐PBMA burger exhibits a compressed, uniform, gel‐like structure consisting of a brown mixture of globular proteins, oils, cellulose, methylcellulose structural binding polymers, salts, and flavorings. With these visual and compositional characteristics in mind, this study explores the potential of TD‐NMR relaxation technology to evaluate the structure of Soy‐PBMA burger products and assess their similarity to the structural profile of real Angus meat burgers.

Angus meat burger and Soy‐PBMA burger samples were analyzed by the TD‐NMR system, and a large‐scale database was generated (see Supporting Information). Fifteen replicates of 1D T_1_ inverse recovery and T_2_ CPMG decay curves of each raw burger group are presented in Figure 1. The pattern of relaxation graphs of all replicates of Angus meat burger samples looks much more uniform in comparison to the graph of processed raw Soy‐PBMA burger samples. The first phase of the relaxation test (up to 500 ms) is shown in the zoomed‐in in of each of the graphs. In all tested burgers, T_1_ of the Angus‐burgers recovery pattern is slower compared to Soy–PBMA burgers. An opposite relaxation pattern can be seen in the graph of T_2_ decay. In all tested burgers, T_2_ of Soy‐PBMA burgers decay pattern is slower compared with Angus burgers. In the second phase of the T_1_ and T_2_ relaxation up to the end of the test (3000 ms), the relaxation rate shows minimal differences if et al for both types of burgers.

### 3.1 Analysis of T₁ Spectra for Microstructural Characterization

**FIGURE 2:**
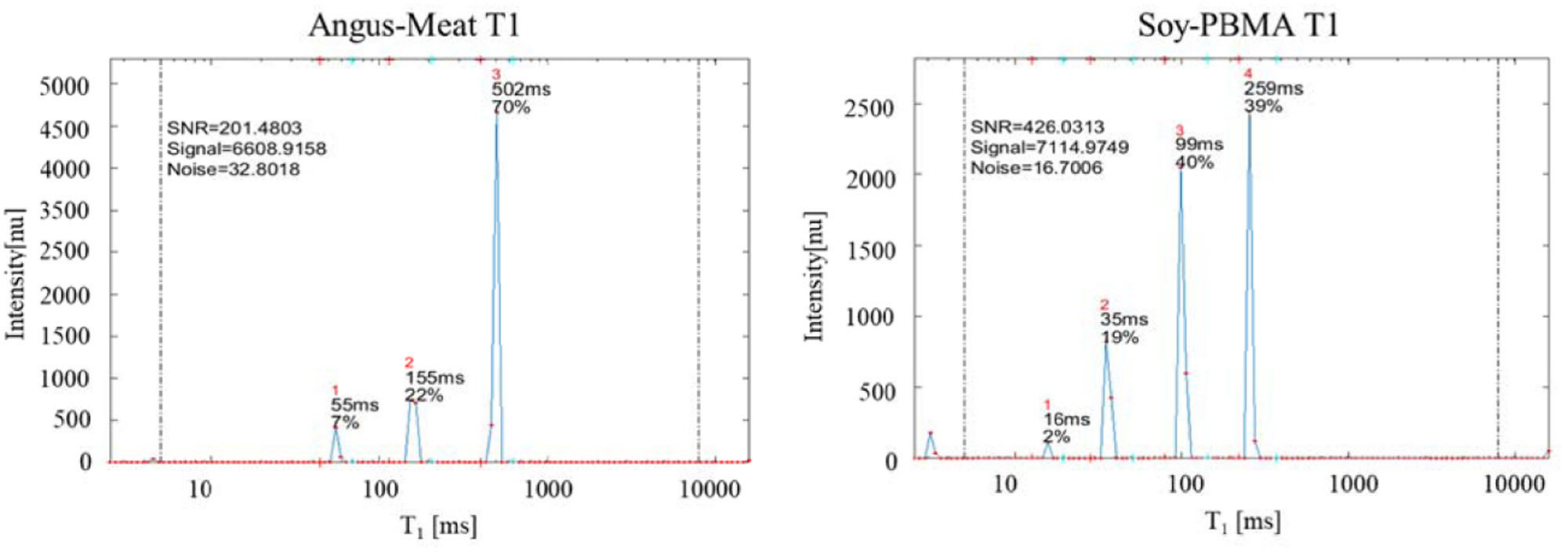
Representative 1D T₁ recovery spectra of Angus meat and Soy‐PBMA burgers. These typical recovery profiles were selected from a database comprising 64 replicates. The main T₁ peak for the Angus meat burger appears at 502 ms, accounting for approximately 70% of the total signal intensity. In contrast, the Soy‐PBMA burger exhibits two major T₁ peaks: one at 99 ms (40%) and another at 259 ms (39%), reflecting the heterogeneous relaxation behavior of its plant‐based components.

**FIGURE 3:**
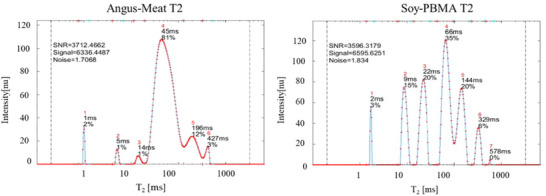
Representative 1D T₂ decay spectra of Angus meat and Soy‐PBMA burgers. These typical decay profiles were selected from a comprehensive database comprising 64 replicates. The main T₂ peak for the Angus meat burger appears at 45 ms and constitutes approximately 81% of the total signal intensity. In contrast, the Soy‐PBMA burger exhibits three major T₂ peaks at 22 ms (20%), 66 ms (35%), and 144 ms (20%), reflecting a more with its plant‐based components.

To gain deeper insight into the microstructural differences between the two burger types, TD‐NMR T₁ and T₂ spectra are presented in Figures 2 and 3, respectively. In the T₁ spectrum of the Angus meat burger (Figure 2), the main peak, corresponding to proton population recovery primarily from myofibrillar proteins, appears at 502 ms and accounts for approximately 70% of the total T₁ signal intensity. In contrast, the Soy‐PBMA burger displays two dominant T₁ peaks associated with proton recovery from processed globular plant proteins: one at 99 ms (40%) and another at 259 ms (39%). Additional minor peaks, representing proton populations associated with rigid polymeric structures, appear at shorter T₁ values in both burger types.

These representative T₁ spectra—also included in the study's database (see Supporting Information)—demonstrate that the T₁ recovery time of Angus meat burgers is significantly longer than that of Soy‐PBMA burgers, indicating differences in molecular mobility and structural composition.

### 3.2 Analysis of T₂ Spectra for Microstructural Characterization

The T₂ spectrum provides further insight into the microstructural organization of the two burger types (Figure 3). In the Angus meat burger, the main T₂ peak—representing proton relaxation associated with the uniform fibrillar arrangement of muscle proteins—appears at 45 ms and accounts for approximately 81% of the total T₂ signal intensity. In contrast, the Soy‐PBMA burger displays a more complex relaxation profile, with two main T₂ peaks: One at 22 ms (20%) and another at 66 ms (35%). These peaks are associated with proton populations in processed globular plant proteins, indicating a broader distribution of mobility states. Additionally, both burger types exhibit minor fast‐relaxing peaks (at 14 ms or shorter T₂ values), likely corresponding to rigid proton populations in structural binding polymeric components—collagen in Angus meat and cellulose/methylcellulose in Soy‐PBMA. Two slow‐relaxing peaks can also be observed: at 196 and 427 ms in the Angus meat burger, and at 144 and 329 ms in the Soy‐PBMA burger. These peaks correspond to the segmental motion of fatty acids.

These representative T₂ spectra, drawn from a dataset of 64 replicates, clearly show that the dominant T₂ relaxation in Angus meat is shorter and more narrowly distributed than in the Soy‐PBMA burger, reflecting fundamental differences in protein structure and molecular organization.

### 3.3 Water Content and Diffusion Properties of Angus Meat and Soy‐PBMA Burgers

A comparison of the total water content and centrifuge‐released water in Angus meat and Soy‐PBMA burgers is presented in Table 1. The total water content, gravimetrically measured from oven‐heated raw samples, was 68.9% and 60.8% by weight for Angus meat and Soy‐PBMA burgers, respectively. Bound water content, determined gravimetrically by low‐speed centrifugation (1500 rpm) of raw burger samples, was 13.8% for Angus meat and 9.3% for Soy‐PBMA burgers.

In addition, the self‐diffusion coefficients (*D*) of water in the same raw burger samples, before oven heating and centrifugation, were measured using the pulsed‐field gradient spin‐echo (PFGSE) method via TD‐NMR (Table 1). The diffusion coefficients were 1.46 × 10⁻⁹ m^2^/s for Angus meat and 1.37 × 10⁻⁹ m^2^/s for Soy‐PBMA burgers.

### 3.4 2D T₁–T₂ Fingerprint Analysis of Angus Meat and Soy‐PBMA Burgers

**FIGURE 4:**
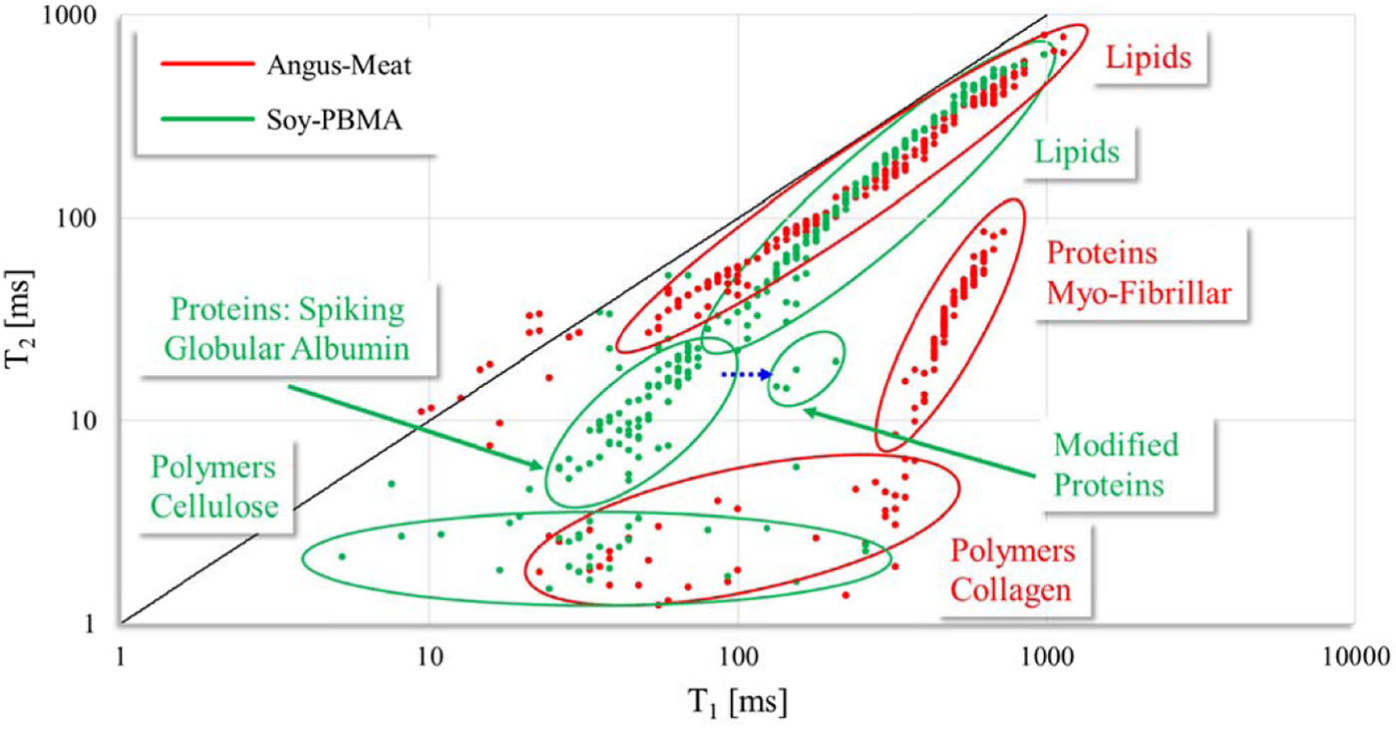
Comparison of 2D T₁‐T₂ relaxation fingerprints of main components in Angus meat and Soy‐PBMA burgers. Thirty samples from each burger type were analyzed. Three major component groups—proteins, oils, and structural polymers—are identified. Albumin and soybean protein isolate spikes were used to locate Soy‐PBMA globular proteins. Mapping of Soy and Angus oils is based on the characteristic T₁ = T₂ diagonal. Plant cellulosic and starch structural polymers in Soy‐PBMA and collagen structural polymers in Angus meat were assigned based on their size and rigidity.

To better visualize and understand the microstructural differences between the two burger types, two‐dimensional (2D) T₁–T₂ fingerprints from thirty measurements of Angus meat burgers were overlaid with thirty measurements of Soy‐PBMA burgers, as shown in Figure 4. Proton populations corresponding to major burger components—such as proteins, oils, and polymers—are identified and labeled for each sample type.

As expected, the proton populations of oils in the tested burgers are distinctly observed along the diagonal line of the T₁–T₂ plot, with only minor differences between Angus meat (dominated by saturated fatty acids) and Soy‐PBMA burgers (dominated by unsaturated fatty acids). Similarly, the proton populations in the lower T₂ range (<5 ms) along the T₁ scale (5–500 ms), associated with rigid structural polymers—collagen and elastin polypeptides characterized by a stiffness gradient from rigid to compliant in Angus meat, and variable rigid and amorphous cellulose and methylcellulose in Soy‐PBMA—show minimal differences.

The main distinction in the T₁–T₂ fingerprints lies between the natural myofibrillar proteins of Angus meat and the processed globular proteins of Soy‐PBMA. Angus meat proteins are concentrated in a T₂ range of 5–100 ms and a T₁ range of 500–800 ms. In contrast, based on spiking tests using globular albumin standards and soybean protein isolate—both separately and in combination with Soy‐PBMA samples—the proton populations assigned to proteins in Soy‐PBMA are distributed over a T₂ range of 5–90 ms and a notably shorter T₁ range of 50–250 ms. Additionally, an extra population of modified proteins in Soy‐PBMA is observed at T₂ values of 7–30 ms and T₁ values of 100–400 ms.

### 3.5 Microscopy Analysis of Burger Microstructure

**FIGURE 5:**
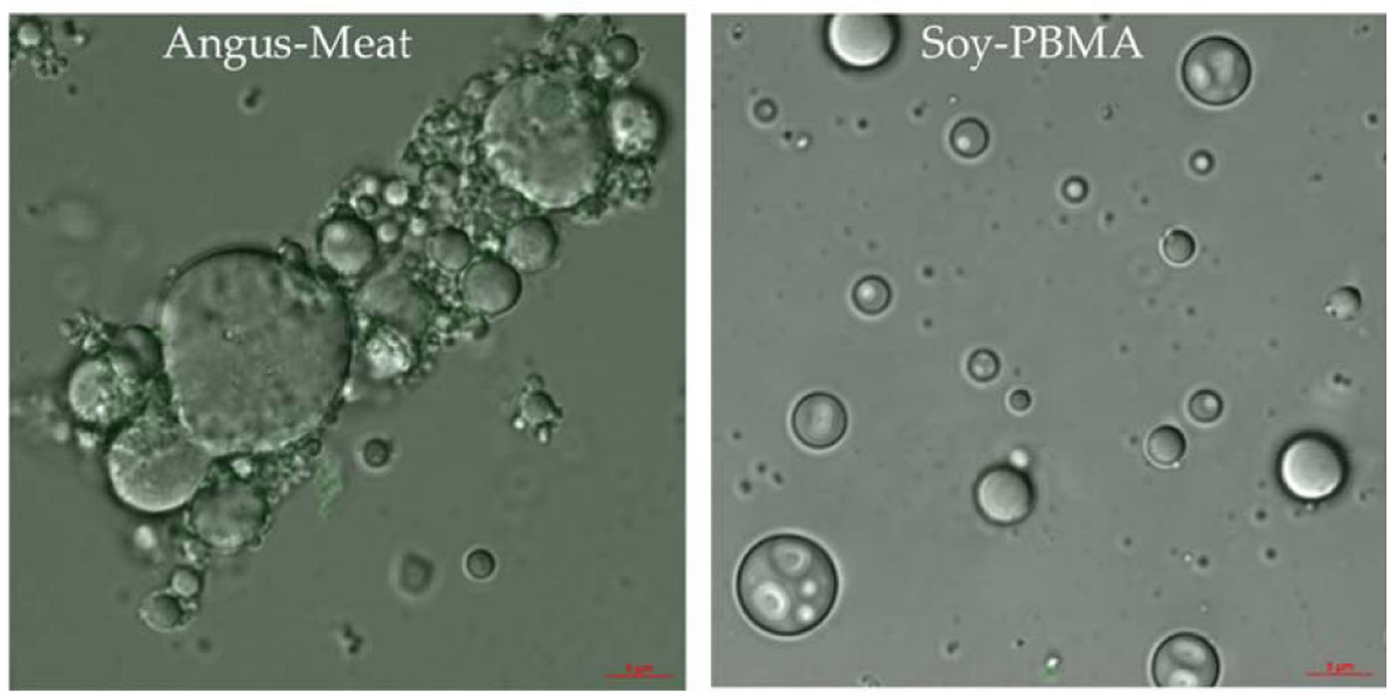
Confocal laser scanning microscopy (CLSM) images of Angus meat and Soy‐PBMA burger samples. The Angus meat burger exhibits an aggregated, bundle‐like structure with a broad distribution of spherical particle sizes. In contrast, the Soy‐PBMA burger displays a well‐dispersed pattern of emulsified spherical vesicles with varying sizes. Samples were diluted 1:10 and imaged at 63× magnification using super‐resolution CLSM to highlight microstructural differences between the two burger types.

To gain further morphological insights, CLSM was performed on both burger types, with representative images shown in Figure 5. The Soy‐PBMA burger samples exhibit a well‐dispersed pattern of emulsified spherical vesicles with variable particle sizes. In contrast, the Angus meat burger samples display an aggregated, undefined bundle‐like structure with a broad distribution of spherical particle sizes.

Microscopy revealed distinct structural differences between the Angus meat and Soy‐PBMA burgers. The Angus burger displayed densely packed, aligned muscle fiber bundles, with fat appearing as globular inclusions distributed between fibrous layers—forming a compact, anisotropic structure characteristic of natural muscle tissue. In contrast, the Soy‐PBMA burger exhibited a porous, isotropic matrix, in which fat was emulsified into uniformly dispersed droplets embedded within a restructured soy protein network. Water was retained primarily within the pores of this sponge‐like plant matrix, whereas in meat it was more tightly bound to fibrous proteins and intracellular compartments. While this microscopy‐based comparison offers valuable insights into microstructural organization, we fully acknowledge the limitation of not using targeted fluorescent dyes, such as Nile Red for lipids or Fast Green for proteins, which would enable more precise differentiation of protein and fat components.

## 4 Discussion

This study evaluates the effectiveness of nondestructive, rapid TD‐NMR relaxation technology for characterizing internal chemical, morphological, and microstructural differences between traditional meat burgers and plant‐based meat analogs.

Visual and microscopic comparisons between Angus meat and Soy‐PBMA burgers revealed substantial structural distinctions that influence key quality attributes, including texture, water retention, and mouthfeel. Water plays a pivotal role in shaping the internal organization of both burger types, influencing molecular interactions and structural integrity (Dekkers et al. 2018; Schreuders et al. 2021; Hu et al. 2024; Kuijpers et al. 2024). Accordingly, special emphasis was placed on quantifying differences in water content and distribution to support the interpretation of structural and relaxation data.

In line with previous studies (De Marchi et al. 2021; Kyriakopoulou et al. 2021), our data demonstrate that the total water content of Angus meat burgers is approximately 8% higher than that of Soy‐PBMA burgers (Table 1). Furthermore, the Angus burgers exhibit a greater proportion of bound water, which is corroborated by their higher measured diffusion coefficient (*D*). This elevated *D* value—reflecting greater proton mobility—is known to correlate with increased water content and less restricted molecular environments in meat matrices (Trujillo et al. 2007).

Understanding both total and mobile water fractions is essential for interpreting TD‐NMR relaxation behavior and resulting structural fingerprints. Water content and dynamics significantly influence T₁ and T₂ relaxation times in TD‐NMR, as these parameters reflect the interaction of hydrogen nuclei, primarily from water, with their molecular surroundings (Bertram et al. 2002; Sørland et al. 2004; Peters et al. 2016; Dekkers et al. 2018; Acri et al. 2021; Hu et al. 2024; Kuijpers et al. 2024).

To aid interpretation, we first analyze the T₁ and T₂ relaxation data separately, followed by an integrated discussion that synthesizes TD‐NMR findings with microscopy, moisture analysis, and diffusion measurements. This approach enables a comprehensive understanding of the structural differences between Angus meat and Soy‐PBMA burgers and highlights the unique capabilities of TD‐NMR for food structure profiling.

### 4.1 T₁ and T₂ Relaxation Analysis and Interpretation

T₁ relaxation time reflects the rate at which excited protons realign with the external magnetic field and is primarily influenced by the macromolecular environment and water mobility. When comparing the T₁ recovery behavior between high‐moisture Angus meat burgers and the relatively lower‐moisture Soy‐PBMA burgers, as indicated by the diffusion coefficient (*D*) and total water content in Table 1, the state and quantity of water in each system emerge as dominant factors. Prior studies have shown that samples with higher moisture content tend to exhibit longer T₁ values, due to enhanced water mobility and reduced molecular restrictions (Marigheto et al. 2008; Bertram et al. 2002). In contrast, lower‐moisture or denser matrices, such as those in processed plant‐based products, often restrict water movement, resulting in shorter T₁ recovery times.

Consistent with these observations, our results demonstrate that T₁ recovery in the high‐moisture Angus meat burger is slower than in the lower‐moisture Soy‐PBMA burger (Figure 1). This trend is also evident in the time‐domain 1D T₁ fingerprint spectra (Figure 2). In Angus meat, a dominant T₁ peak at 502 ms accounts for approximately 70% of the signal, corresponding to protons associated with natural myofibrillar proteins. In contrast, the Soy‐PBMA burger exhibits two major T₁ peaks at shorter recovery times—99 ms (40%) and 259 ms (39%)—indicating more restricted water mobility within its processed, globular plant protein matrix. This contrast highlights fundamental differences in protein structure and water interaction: The single dominant peak in Angus meat suggests a relatively uniform myofibrillar architecture, while the dual peaks in Soy‐PBMA reflect its heterogeneous, restructured protein environment.

Additional smaller T₁ peaks in both samples represent rigid structural polymeric components of different origins—collagen and elastin in Angus meat, and cellulose and methylcellulose in Soy‐PBMA. The relaxation behavior of these polymers depends on factors such as molecular rigidity, cross‐linking, hydration level, and crystallinity (Wiesman et al. 2018; Alexandretti et al. 2019). For instance, collagen, with its triple‐helical structure and hydrogen bonding network, exhibits longer T₁ values due to limited molecular motion, while crystalline cellulose, a plant‐derived polymer, shows shorter T₁ values that predominantly reflect surface hydration.

In contrast, T₂ relaxation time captures how quickly proton spins lose phase coherence due to local magnetic field inhomogeneities (Bertram et al. 2002). It is particularly sensitive to molecular motion and microstructural rigidity. Interestingly, T₂ relaxation in the high‐moisture Angus meat burger decayed faster than in the lower‐moisture Soy‐PBMA burger (Figure 1). This finding aligns with prior work suggesting that T₂ is influenced more by structural environments—including pore confinement, protein packing, and water binding capacity—than by total moisture alone (Peters et al. 2016; Dekkers et al. 2018; Hu et al. 2024).

The 1D T₂ fingerprint spectra (Figure 3) revealed greater complexity than the corresponding T₁ profiles, with six distinguishable peaks in comparison to four. In the Angus burger, the dominant T₂ peak occurs at 45 ms and represents approximately 81% of the signal, corresponding to water bound within myofibrillar structures. In Soy‐PBMA, two major T₂ peaks are observed at 22 ms (20%) and 66 ms (35%), reflecting water located in regions with varying degrees of molecular restriction within the plant protein matrix. This multi‐peak pattern reflects a heterogeneous internal structure with a range of pore sizes, consistent with a gel‐like matrix, rather than the dense, aligned fibrillar organization typical of animal muscle tissue.

Smaller peaks with T₂ values below 14 ms in both burger types are attributed to rigid, crystalline, and insoluble structural polymers. In Angus meat, these signals are linked to collagen, which exhibits moderate T₂ decay due to its semi‐rigid, triple‐helical architecture. In the Soy‐PBMA burger, the short T₂ signals are associated with cellulose and methylcellulose—rigid plant‐based components that exhibit minimal water interaction beyond surface hydration (Wiesman et al. 2018; Besghini et al. 2019).

Finally, T₂ peaks with longer relaxation times (144–429 ms), consistent across both burger types, are attributed to the segmental motion of fatty acids, particularly triacylglycerols (Resende et al. 2019, 2020). These signals remained unchanged in dry burger samples after oven drying (data not shown), indicating their independence from water content. Differences in T₂ values within this range may reflect varying degrees of fatty acid saturation, with Angus burgers typically richer in saturated fats and Soy‐PBMA products containing more unsaturated fatty acids (Kyriakopoulou et al. 2021; Konrad et al. 2024).

### 4.2 2D T₁–T₂ Relaxometry Fingerprinting: Structural Insights

The two‐dimensional (2D) T₁–T₂ proton NMR relaxometry fingerprinting map offers a powerful, non‐destructive method for characterizing distinct water populations within complex food matrices (Acri et al. 2021; Colnago et al. 2021). This technique enables the differentiation of free, loosely bound, and tightly bound water—key parameters for understanding internal water dynamics in high‐moisture systems like Angus meat burgers, compared to lower‐moisture alternatives such as Soy‐PBMA burgers.

The data presented in Figure 4 represent cumulative results from multiple TD‐NMR scans, with overlaid T₁–T₂ spectra for both Angus meat and Soy‐PBMA burger samples. This composite approach enhances confidence in the reproducibility and statistical significance of the observed spectral patterns. The spatial separation of protein‐related proton populations—signals from Angus meat aligning along the right‐hand side of the T₁ axis, and those from Soy‐PBMA clustering in the lower‐left quadrant below the diagonal—demonstrates a clear distinction in water–protein interaction profiles. These findings reinforce conclusions drawn from 1D T₁ spectra, confirming that water interacts differently with myofibrillar proteins in meat than with globular plant proteins in Soy‐PBMA formulations.

Analysis of the same protein‐associated signals along the T₂ axis reveals a more modest differentiation between the two burger types, consistent with trends observed in the 1D T₂ data. This pattern aligns with previous studies (Bertram et al. 2002; Sørland et al. 2004; Dekkers et al. 2016) and suggests that the most pronounced structural differences are captured along the T₁ dimension, which is more sensitive to hydration dynamics and protein conformational states. Collectively, these findings support the interpretation that water in Angus meat is more strongly associated with intracellular compartments and tightly packed myofibrillar bundles, whereas in Soy‐PBMA, water is primarily associated with the surfaces of emulsified vesicles, mediates intercellular interactions, and occupies interstitial spaces within the porous plant–protein matrix (Silletta et al. 2016).

Notably, the 2D T₁–T₂ spectra also reveal the presence of an additional minor protein population in the Soy‐PBMA samples, characterized by relatively longer T₁ values. This signal may reflect partially structured, fiber‐like proteins formed during the texturization and hydration steps common in plant‐based meat analog processing (Greer et al. 2018).

Fat‐associated signals, primarily from triacylglycerols, appear along the T₁ = T₂ diagonal, consistent with the well‐established equivalence of T₁ and T₂ relaxation times in oil systems (Berman et al. 2013; Resende et al. 2019, 2020, 2021). Differences in the distribution of these lipid‐related signals between Angus meat and Soy‐PBMA likely reflect variations in fatty acid composition, with Angus meat being richer in saturated fats and Soy‐PBMA composed primarily of unsaturated fatty acids (Resende et al. 2021; Osheter et al. 2022, 2023; Konrad et al. 2024).

Structural polymeric components, which are rigid and poorly hydrated, exhibit rapid T₂ relaxation and cluster at the lower end of the T₂ axis. However, these components are more widely distributed along the T₁ axis due to differences in molecular size and rigidity. Collagen and elastin in Angus meat, being larger and more elastic, appear toward the right (longer T₁ values), whereas cellulose and methylcellulose in Soy‐PBMA—smaller, more rigid polymers—are shifted to the left. These observations are consistent with prior reports on water–polymer interactions (Marigheto et al. 2008; Wiesman et al. 2018).

The complementary techniques presented in this study contribute to a deeper understanding of the structural properties of the two burger types. It is important to note the scale gap between the methods: visual and microscopy analyses operate at the macro‐ and microscale levels, providing qualitative insights into overall morphology, fiber alignment, and pore structure; whereas TD‐NMR operates at the molecular level, probing water–protein interactions, molecular mobility, and microstructural confinement. To bridge this scale gap and explain how features observed visually are reflected in TD‐NMR relaxation behavior, specifically in the distribution and relaxation times (T₁ and T₂) of different water populations, we propose that integrating multiscale data enables a more comprehensive and robust interpretation of food structure.

### 4.3 Integrated Multiscale Interpretation

The complementary techniques employed in this study offer a deeper, multiscale understanding of the structural properties of the two burger types. Visual and microscopy analyses provide qualitative insights at the macro‐ and microscale levels, revealing differences in morphology, fiber alignment, and pore architecture. In contrast, TD‐NMR relaxometry probes the molecular scale, capturing information about water–protein interactions, molecular mobility, and the extent of microstructural confinement. While each technique provides valuable information on its own, the integration of these multiscale datasets allows for a more comprehensive and robust interpretation of food structure. Specifically, this approach helps bridge the scale gap by linking features observed visually—such as fat droplet dispersion and matrix porosity—to differences in T₁ and T₂ relaxation behaviors, which reflect the mobility and distribution of distinct water populations.

We acknowledge, however, that the lack of targeted fluorescent dyes—such as Nile Red for lipids or Fast Green for proteins—limits our ability to definitively identify specific structural components within the CLSM images. This limitation is now explicitly addressed, and we highlight the potential of future work to incorporate such staining techniques to further enhance chemical specificity and structural interpretation.

### 4.4 Implications for Food Structure Profiling

Structural profiling of both meat and plant‐based meat analogs is critical for advancing product quality, functionality, and consumer acceptance. The internal architecture of a food product directly influences key attributes such as texture, moisture retention, visual appeal, and overall mouthfeel. For both traditional meat and plant‐based burgers, understanding how proteins, fats, and polymeric components are organized and interact within the matrix enables targeted improvements in juiciness, bite, chewability, and overall sensory experience.

Detailed structural characterization also enables the correlation of microstructural features—such as muscle fiber alignment, fat globule dispersion, and gel matrix formation—with consumer sensory feedback. This information can guide formulation strategies by identifying plant‐based ingredients that most effectively replicate meat‐like qualities. Furthermore, it supports iterative product optimization and ensures batch‐to‐batch consistency, which is essential for maintaining brand identity, quality control, and compliance with regulatory and nutritional standards.

By integrating TD‐NMR relaxometry data with complementary techniques such as visual microscopy, diffusion analysis, and gravimetric moisture quantification, this study presents a comprehensive, science‐based framework for differentiating and characterizing the structural and hydration dynamics of both real meat and plant‐based meat systems. This approach offers valuable insights for product development, quality assurance, and the future design of high‐performance meat analogs.

## 5 Summary

Microscopy and visual analysis revealed clear morphological differences between Soy‐PBMA and Angus meat burgers. The Soy‐based burgers exhibited a gel‐like, porous matrix formed by restructured plant proteins, while the Angus burgers displayed a more defined, fibrous architecture characterized by aligned muscle bundles. These structural differences contribute to the distinct texture and mouthfeel observed between the two products.

Moisture analysis showed that Soy‐PBMA burgers contain less total water than Angus meat burgers. Additionally, water release testing under centrifugation indicated that Angus burgers possess a higher water‐binding capacity. This suggests stronger intracellular water interactions within the fibrous meat matrix, in contrast to the interstitial, porous interactions observed in the Soy‐PBMA matrix.

These conventional measurements provided foundational insights that align with and reinforce findings obtained through TD‐NMR relaxometry. From the initial acquisition of T₁ and T₂ relaxation signals, it was evident that the internal microstructures of the two burger types differ substantially. The 1D T₁ and T₂ spectra confirmed these distinctions, while 2D T₁–T₂ relaxation fingerprinting offered a more nuanced structural profile. The primary structural divergence lies in the relaxation behavior of myofibrillar proteins in Angus meat versus the processed globular proteins in Soy‐PBMA—differences that directly correspond to the observed morphological contrasts.

Taken together, these findings confirm that the objectives of this study were successfully achieved. TD‐NMR relaxometry proved to be a rapid, non‐destructive, and reliable technique for differentiating and profiling the internal structure of burger products derived from different sources and processing methods. These applications of TD‐NMR offer valuable support for the development of improved plant‐based alternatives and help ensure quality and consistency in commercial production.

## 6 Conclusions

TD‐NMR relaxometry—including 1D and 2D T₁–T₂ mapping and diffusion coefficient (*D*) analysis—provides powerful, non‐destructive insights into the internal microstructure of food products. These techniques generate unique structural fingerprints for both Soy‐PBMA and Angus meat burgers, offering a robust framework for evaluating structural similarity and assessing how effectively plant‐based products replicate the characteristics of real meat.

The ability of TD‐NMR to quantify key parameters such as water mobility, protein–water interactions, and fat distribution makes it a highly valuable tool for food quality assurance, formulation development, and innovation. Looking forward, TD‐NMR applications may be expanded to monitor textural integrity, moisture dynamics, and structural stability over time, critical factors influencing shelf life, consumer experience, and product consistency. As such, TD‐NMR has the potential to play a central role in optimizing the development and standardization of next‐generation plant‐based foods.

## Author Contributions

**Zeev Wiesman**: conceptualization, investigation, writing original draft, formal analysis, project administration, supervision, funding acquisition, resources, writing–review and editing. **Moshe Hai Azachi**: investigation, methodology, visualization. **Tatiana Oshether**: investigation, methodology, validation, visualization, software, data curation.

## Conflict of Interest

The authors declare no conflicts of interest.

## Supporting information




**Supporting Material**: jfds70376‐sup‐0001‐SuppMat

**Supporting Material**: jfds70376‐sup‐0002‐SuppMat

**Supporting Material**: jfds70376‐sup‐0003‐SuppMat

**Supporting Material**: jfds70376‐sup‐0004‐SuppMat

**Supporting Material**: jfds70376‐sup‐0005‐SuppMat

## Data Availability

No data were used for the research described in the article.
